# Mir-55 inhibition can reduce cell proliferation and induce apoptosis in Jurkat (Acute T cell Leukemia) cell line

**Published:** 2014-12-10

**Authors:** Sh Alizadeh, S Kaviani, M Soleimani, S Abroun, Z Kashani-Khatib, A Asgharzadeh, H Dargahi, R Mousavi

**Affiliations:** 1 Department of Hematology, Allied Medical School, Tehran University of Medical sciences, Tehran, Iran.; 2 Department of Hematology, Tarbiat Modares University, Tehran, Iran.; 3 Department of Hematology, Mashad University of Medical sciences, Mashhad, Iran.; 4 Department of Health care services, Medical School, Tehran University of Medical sciences, Tehran, Iran.

**Keywords:** miRNA, miR-155 inhibitor, Apoptosis, jurkat cells.

## Abstract

**Background:**

MicroRNAs are small and non-coding RNA molecules with approximately 22 nt in length that cause inhibition of translation or degradation of mRNA. MiR-155 is a kind of molecule with different functions, such as its role in proliferation, apoptosis, inflammation, differentiation, and immunity. One of its best known functions is apoptosis that affects on caspase-3 activity. The main aim of this study was evaluation of miR-155 inhibition effect on cell proliferation and apoptosis induction in Jurkat cells.

**Material and Methods:**

In this study, Jurkat cells along with MTT assay were used for evaluation of sensitivity to varied concentrations of miR-155 inhibitor (25, 50 and 75 nmol). MiR-155 expression level was analyzed using the quantitative real-time polymerase chain reaction (QRT-PCR). Caspase-3 activity was measured by caspase-3 colorimetric activity assay kit. Unpaired t-test was applied for the analysis of MTT and apoptosis results. Probability of 5% was assumed as statistically significant.

**Results:**

According to our results, the use of miR-155 inhibitor increased the activity of caspase-3 by 2 fold in 75 nmol concentration. In this research, we found that the proper increase of miR-155 inhibitor concentration can inhibit miR-155 and consequently increase caspase-3 activity and induce apoptosis in the Jurkat cells leading to cell death ultimately.

**Conclusions:**

Apoptosis induction by miRNAs activation or inhibition is probably one of the best and low risk ways of cell death induction in malignancies. Due to role of miR-155 in several cancer cells, it may be used as a therapeutic target in future.

## Introduction

MicroRNAs are small, single strand and non-coding molecules with conserved sequence containing approximately 22 nt in length, which are encoded in eukaryote cells. They act as post-transcriptional regulators and cause inhibition of translation or degradation of mRNA (1-5) by binding to 3′UTR of target genes (6-8). In mammals, it is suggested that more than 60% of all protein coding genes are controlled by miRNAs (9). These small molecules have different roles in different cell types including proliferation, differentiation , and apoptosis (10). The expression pattern of miRNAs is different in normal and cancer tissues and due to this issue many miRNAs are deregulated in cancer. These small molecules have different functions in cancer such as tumor suppression or oncogenic effect (10, 11), cell cycle regulation (12,13), p53 pathway regulation (14-16), apoptosis induction or tolerance (17,18), invasion and metastasis (19), and angiogenesis (20,21). 

One of the best known roles of miRNAs in cancer is down-regulation of miR-15a and miR-16-1 in chronic lymphoid leukemia (CLL) (22). Deregulation of miRNAs is related with several types of tumors including colon, breast, lung , and other cancers (23, 24). One of the miRNAs that is deregulated frequently in some cancers is miR-155. Overexpression of miR-155 has been found in a number of cancers, suggesting that this miRNA is involved in carcinogenesis (25). MiR-155 is a molecule with wide variety of functions including hematopoiesis, immunity, and inflammation. MiR-155 has also an essential role in lymphoid development, myelopoiesis, and erythropoiesis (26-28). Expression of miR-155 increases in lymphoid and it decreases in myeloid and erythroid developments (29, 30). MiR-155 has an essential role in B and T cells developmental process including isotype switching, high affinity antibody production in B cells , and the fate of Th1 or Th2 determination in T cells (31-34). In inflammation, lipopolysaccharide of bacteria (LPS) and other inflammatory mediators such as IFN-β and TNF-α can induce miR-155 expression in the macrophage and monocyte cells (35, 36). Overexpression of miR-155 has been found in leukemia such as AML (37, 38) and some solid tumors including colon cancer (23, 24), cervical cancer (39), thyroid carcinoma (40) , and B cell lymphoma (41-43). 

Apoptosis or programmed cell death is a physiological self-destruction process that is controlled by different genes expression. Proliferation and apoptosis are two essential factors in tissue homeostasis and imbalance of them that may give rise to cancer. Some of miRNAs have roles in tumor through apoptotic or anti-apoptotic effect. Among them miR-155 plays a significant role in cell growth and apoptosis (44-46). This study aimed to evaluate the effect of miR-155 in cell death or apoptosis in Jurkat cells.

## Materials and methods

**Cell culture**

Jurkat cell line was supplied by the National Cell Bank of Iran, Pasteur Institute, Tehran, Iran. These cells were grown in RPMI1640 medium (Gibco.USA) with 10% fetal Bovine serum (FBS) (Gibco.USA), 1X Penicillin streptomycin antibiotic , and 2mM L-glutamine at 37ºC under a humidified atmosphere consisting of 95% air and 5% CO2 for 7 days. In the day of transfection, trypan-blue was used for cells counting and viability determining. Then 2×10^5^ cells per well were seeded in 3 ml complete growth medium in a 6-well culture plate.

**Oligonucleotide sequences**

**Fluorescent inhibitors:** anti-miR-155(5'-CCTATCACGATTAGCATT-3', EXIQON, product no: 410078-04); Fluorescent scramble siRNA (5'-GTGTAACACGTCTATACGCCCA-3', EXIQON, product no: 199004-08). 

**QRT-PCR primers:** miR-155 forward primer (5'-TTAATGCTAATCGTGATAGG-3'), U6 forward primer (5'-CTCGCTTCGGCAGCACACATATAC-3'), U6 reverse primer (5'-ACGCTTCACGAATTTGCGTGTC-3').

**MiRCURY LNA™ microRNA inhibitor and scramble transfection**

MiRCURY LNA™ microRNA inhibitor was purchased from Exiqon (EXIQON, Denmark). Lipofectamine 2000 (Invitrogen, USA) was used for Jurkat cells transfection according to the manufacturer’s manual. Briefly one day before transfection, 2×10^5^ cells were plated in 2.5ml medium without antibiotic and plus 10% FBS. In one tube, 100 nmol miR-155 inhibitor was diluted in 250µl OPTIMEM (Gibco, USA). In another tube, 5µl Lipofectamine 2000 was diluted in 250µl OPTIMEM. After 5minutes incubation, two tubes were combined and incubated for 20 minutes. Finally, this compound was added to the cultured plates. Transfection of the scramble was done by a similar manner. In this study scramble was used for the evaluation of transfection efficiency and non-specific effects of oligonucleotides. All tests were done in duplicate.

**RNA extraction**

Total RNA was extracted from the control, scramble and inhibitor treated samples containing 2×10^5^ cells (from 6-well culture plate) by using Biozol reagent in the third and seventh days post transfection. Briefly, 500μl biozol was added to the cells and followed by phenol/cholorophorm precipitation according to the manufacturer’s instructions. The purity examination of the extracted RNA was done spectrophotometrically at 260/280nm and gel electrophoresis.

For gaining maximum purity, the extracted RNA was treated by DNAse I (fermentas) based on the manufacturer’s guidelines.

**MiRNA real time PCR**

In this study, the third day extracted RNA was used for miRNA assay. The High-Specificity miRNA QPCR core reagent kit provided the reagents for quantitative PCR amplification of the cDNA templates derived from the miRNAs within a total RNA population. Because of their short length, miRNAs are difficult to detect through standard QRT-PCR protocols. As the first step, the miRNA 1st-strand cDNA synthesis kit (Stratagene, USA) was used to elongate the miRNAs in a polyadenylation reaction. Then the polyadenylated RNA was reverse transcribed into QPCR-ready cDNA. Then the target of interest was amplified and detected using the high-specificity miRNA QPCR core reagent kit (Stratagene, USA). The universal reverse primer served as the downstream primer in this QPCR reaction, so the specificity of the QPCR reaction was provided by the miRNA-specific forward primer. Our forward primer for detection of miR-155 was 5'-TTAATGCTAATCGTGATAGGGGT-3'.

**MTT assay**

The day after transfection, the culture medium was replaced with fresh RPMI containing 10% FBS. The culture plates were incubated for the next day at 37˚C. Then, the cells were treated with MTT solution (5 mg/ml) for 4 h. Thereafter, dimethyl sulfoxide (DMSO) was added to each well and the absorption was measured at the wavelength of 540 nm by a spectrophotometer.

**Apoptosis assay**

Apoptosis assay was carried out in duplicate experiments using caspase-3 colorimetric activity assay kit that did not have any positive control (Millipore Company). Briefly miR-155 inhibitor transfections were performed as mentioned above. After 72 h of transfection, 0.5×10^6^ cells were suspended in 100µl of chilled 1X cell lyses buffer and incubated on ice for 10 minutes. Then they were centrifuged for 5 minutes at 10000g. Mixtures assay were prepared in a 96-well plate. 20 µl of 5X assay buffer and 10 µl of caspase-3 substrate (Ac-DEVD-pNA) were added to each test sample. The test samples were incubated for 2 hours at 37C. Then absorbance at 405 nm was measured with a microtiter plate reader. Fold increase in caspase-3 activity was calculated by comparing the OD reading from the induced apoptotic test samples with the level of uninduced control.

**Statistical analysis**

Unpaired t-test was performed to investigate the differences in the obtained results of the three groups. Probability of 5% was assumed as statistically significant.

## Results

**Study subgroups, transfection and miRNA assay**

The Jurkat cells were divided into 3 groups: control group, scramble group that was transfected by 75nmol of scramble antisense, as well as test group that was transfected by 25nmol, 50nmol, and 75nmol of miR-155 inhibitor. Transfection efficiency was determined using inverted fluorescence microscope. The transfected cells with at least 80% efficiency were selected for further analysis. QRT-PCR analyses revealed that miR-155 expression was not strongly decreased in the cells transfected by different concentrations of miR-155 inhibitor. No significant differences were observed in miR-155 expression between the control and scramble groups.

**Microscopy**

**Figure 1:**
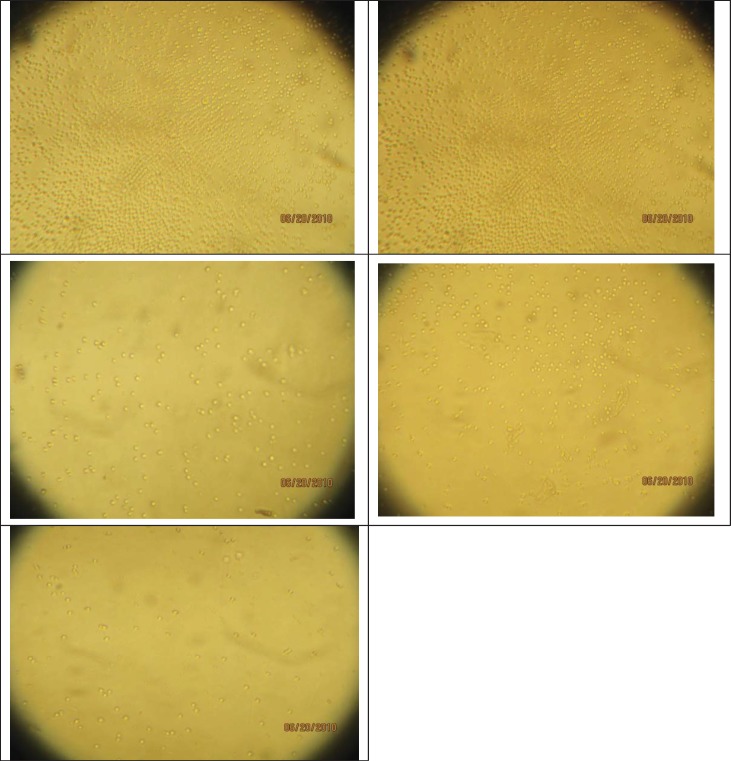
The impact of miR-155 inhibitor on the growth and proliferation of Jurkat cells using light microscopy (100x). (a) Control Jurkat cells. (b) Jurkat cells transfected by 75nmol of scramble. (c) Jurkat cells transfected by 25nmol of miR-155 inhibitor. (d) Jurkat cells transfected by 50nmol of miR-155 inhibitor. (e) Jurkat cells transfected by 75nmol of miR-155 inhibitor.

MiR-155 plays a great role in proliferation of Jurkat cells. In this study, miR-155 inhibitor was used for controlling the cell proliferation. We saw a significant decrease in cell count (by trypan blue staining) and significant cell death increase in the miR-155 inhibitor transfected cells in compare to the control and scramble transfected samples after 48 hours of incubation. Reduction of cell count had direct relation with the added amount of inhibitor. Concentrations of the inhibitors were 25, 50 and 75nmol. By increase of the inhibitor concentration, the cell death rate was increased. By the increase of the inhibitor concentration to 75 nmol, the highest amount of cell death was observed (Fig.1).

**MTT test**

Cytotoxic effects of different concentrations of miR-155 inhibitor and scramble were evaluated 48 hours after transfection by MTT test. Then the optical densities at 540 nm were obtained for all the three groups: 0.568 for the control group, 0.521 for the scramble group, 0.391, 0.303, and 0.218, respectively for the 25nmol, 50nmol and 75nmol concentrations of miR-155 inhibitor. 

**Figure 2:**
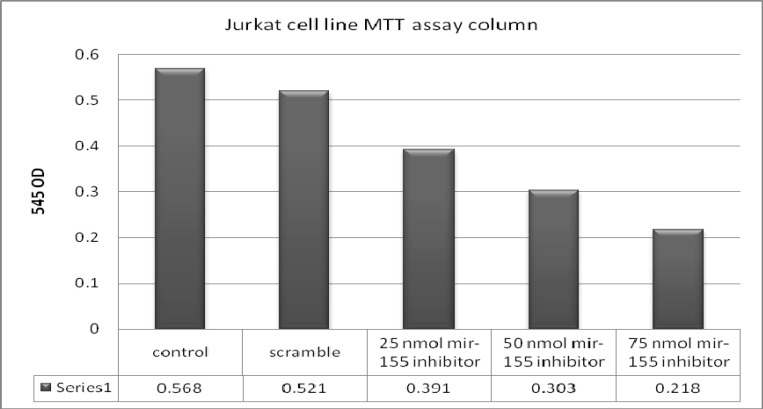
MTT assay on the control, scramble and test groups. OD of the tests is shown in the below row.

These results demonstrated that optical density and cell viability were similar in the control and scramble groups .In fact, there was no statistical difference between control and scramble group (t-test=2.20, p=0.15). However, there were significant differences in the optical density and cell viability between miR-155 inhibitor transfected and control or scramble groups. In other words, there were significant statistical differences among control group; 25nmol, 50 nmol and 75nmol of miR-155 inhibitor transfected groups [(t-test=9.05, p value=0.01), (t-test=4.9, p=0.03) and (t-test=7.06, p= 0.01) respectively)]. As shown in Fig.2 both optical density and cell viability decreased gradually with the increase of the miR-155 inhibitor concentration (0.391, 0.303 and 0.218, respectively). 

At the 75nmol concentration of miR-155 inhibitor, optical density and cell viability were less than half of their value in the control group. 

These data indicated that higher concentrations of miR-155 inhibitor had higher toxicity effect on the Jurkat cells and could decrease their viability and proliferation.

**Caspase-3 activity**

The result also showed that through miR-155 inhibition, activity of the caspase-3 was increased. In the control group, caspase-3 activity was low (0.048) and in the scramble group it was 0.05. In the 50 and 75nmol concentration of miR-155 inhibitor, caspase-3 activity was 0.766 and 0.873, respectively. In the 75nmol concentration of the miR-155 inhibitor, activity of the caspase-3 was increased by 2 fold comparing to the control group. There was no statistical difference between control and scramble group (t-test=0.44, p=0.69).

**Figure 3:**
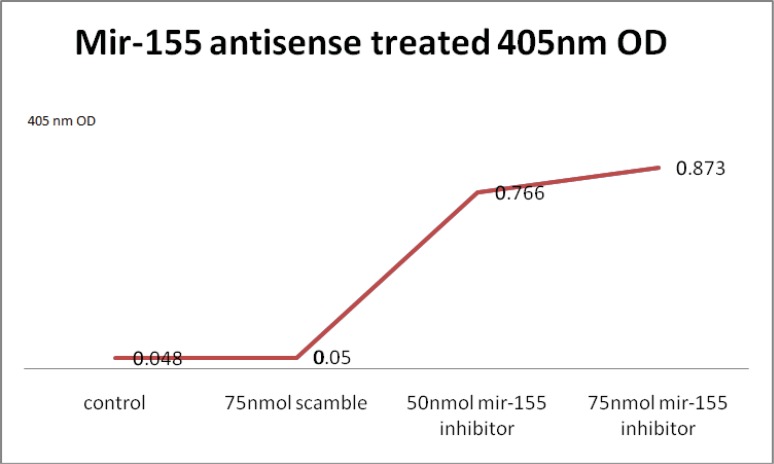
Effect of miR-155 inhibitor on caspase-3 activity in the control, scramble and test groups. Increase in the caspase-3 activity of miR-155 inhibitor transfected Jurkat cells is remarkable.

But according to these data, there were statistically significant differences in optical density and caspase-3 activity among the control; scramble and 50nmol, 75nmol of miR-155 inhibitor transfected groups [(t-test =101.82; p=0.0001) (t-test=117.3, p value=0.0001) respectively] (Fig. 3).

Authors believed that another method such as Annexin V staining or immune blotting for pro- and mature caspase-3 could have been better method for the demonstration of apoptosis.

## Discussion

In most cancers, the use of conventional treatments have many side effects and may well fail to improve disease because of drug resistance (46). Therefore, methods with minimal side effects and rapid recovery are required. New therapeutic strategies in wide variety of malignancies are inhibition of specific target mRNAs (47). The clear functions of miRNAs as tumor suppressor genes (blocking the malignant potential) and oncogenes (activating the malignant potential), revealed their critical role in tumor genesis (48). Since the discovery of the action of miRNAs in the silencing of genes expression, great possibilities have been generated for cancer therapies (49). There are different therapeutic strategies including inhibitor mediated inhibition (oligonucleotides), miRNA replacements , or viral vector encoded miRNAs (50). One of the best methods for therapeutic targets is the use of synthetic anti miRNA oligonucleotides in cancers, which are used both in vivo and in vitro (51-53). Efficacy of this method depends on the effective dose, selection of a right cell, proper time , and stages of malignancies (54). 

Many of researches confer that miRNAs can affect all characteristics of the malignant cells including self-sufficiency in growth signals, insensitivity to anti-growth signals, evasion from apoptosis, limitless replicative potential, angiogenesis, and invasion and metastasis (55). 

The role of different types of miRNAs has been revealed in the regulation of apoptosis. Some of these miRNAs promote apoptosis, whereas others protect against it. Some of the miRNAs act on the survival or death signaling pathways, whereas others affect cell death through mediating apoptotic signals (56). Among the best examples are miR-15 and miR-16 that can induce apoptosis in CLL by blocking survival factors such as BCL-2 and BAD (17).

MiR-155 is a multifunctional molecule that has an important role in different cancers by several mechanisms (57). This miRNA is involved in B-cell malignancies such as Burkitt's lymphoma, CLL, and non- Hodgkin's lymphoma. In addition to lymphoma and lymphoid leukemia, overexpression of miR-155 has been found in bone marrow blast of acute myeloid leukemia (M4 and M5) (37). Some solid tumors including 

thyroid carcinoma (40), colon cancer (24), cervical cancer (39), pancreatic ductal adenocarcinoma (58, 59), lung cancer (60), cardiovascular diseases, and viral infection (61) have also increased expression of miR-155. Several miRNA activities such as miR-155, miR-182 and miR-96 associated with reducing caspase-3 activity (62). 

As mentioned, in our study QRT-PCR analyses revealed that miR-155 expression was not strongly decreased in the cells transfected by different concentrations of miR-155 inhibitor. LNA binds and titrates out the pool of miR-155 but does not cause a degradation in the miRNA.

We suggest a co-transfection of cells with a luciferase reporter with and without a miR-155 binding site as a better method to determine whether the active pool of miR-155 decrease in future studies.

In this study, we suppressed miR-155 activity by miRCURY LNA™ microRNA inhibitor  and observed that caspase-3 activity increased by 2 folds in 75nmol concentration of inhibitor but Ovcharenko and colleagues showed that miR-155 caused suppression of apoptosis in MDA-MB-453 breast cancer and human T cell leukemia Jurkat cells by blocking caspase-3 activity by identification of genes that differentially influence TRAIL-induced apoptosis through siRNA and miRNA library screening.Totally our results were in consistent with the findings of this group (62). 

Induction of apoptosis by miRNAs can cause a decrease in proliferation of malignant cells, resulting in the prevention of their growth progression. Due to role of miR-155 in several cancer cells, miR-155 inhibitors may be used as a therapeutic tool in near future.

Since miR-155 is one of the most widely up regulated miRNA in multiple human tumors and several reports have indicated that miR-155 inhibition can induce apoptosis, use of patients samples instead of cell lines and models can strongly improve implication of this type studies.

Further investigation would be necessary for identification of the exact mechanism through which miR-155 inhibitor influence the growth and proliferation of Jurkat tumor cells.
